# The Evolution of Gene Duplicates in Angiosperms and the Impact of Protein–Protein Interactions and the Mechanism of Duplication

**DOI:** 10.1093/gbe/evz156

**Published:** 2019-07-31

**Authors:** Jonas Defoort, Yves Van de Peer, Lorenzo Carretero-Paulet

**Affiliations:** 1 Department of Plant Biotechnology and Bioinformatics, Ghent University, Belgium; 2 VIB Center for Plant Systems Biology, Ghent, Belgium; 3 Bioinformatics Institute Ghent, Ghent University, Belgium; 4 Department of Biochemistry, Genetics and Microbiology, University of Pretoria, South Africa

**Keywords:** protein–protein interaction, expression divergence, whole genome duplication, small-scale duplication, duplicate retention, angiosperms

## Abstract

Gene duplicates, generated through either whole genome duplication (WGD) or small-scale duplication (SSD), are prominent in angiosperms and are believed to play an important role in adaptation and in generating evolutionary novelty. Previous studies reported contrasting evolutionary and functional dynamics of duplicate genes depending on the mechanism of origin, a behavior that is hypothesized to stem from constraints to maintain the relative dosage balance between the genes concerned and their interaction context. However, the mechanisms ultimately influencing loss and retention of gene duplicates over evolutionary time are not yet fully elucidated. Here, by using a robust classification of gene duplicates in *Arabidopsis thaliana*, *Solanum lycopersicum*, and *Zea mays*, large RNAseq expression compendia and an extensive protein–protein interaction (PPI) network from *Arabidopsis*, we investigated the impact of PPIs on the differential evolutionary and functional fate of WGD and SSD duplicates. In all three species, retained WGD duplicates show stronger constraints to diverge at the sequence and expression level than SSD ones, a pattern that is also observed for shared PPI partners between *Arabidopsis* duplicates. PPIs are preferentially distributed among WGD duplicates and specific functional categories. Furthermore, duplicates with PPIs tend to be under stronger constraints to evolve than their counterparts without PPIs regardless of their mechanism of origin. Our results support dosage balance constraint as a specific property of genes involved in biological interactions, including physical PPIs, and suggest that additional factors may be differently influencing the evolution of genes following duplication, depending on the species, time, and mechanism of origin.

## Introduction

Because of the prominent role attributed to gene duplication in generating evolutionary novelty and adaptation, helping to overcome ecological challenges and contributing to the emergence of relevant agronomic traits, the molecular mechanisms driving the evolutionary and functional fate of genes after duplication have been the object of intense research (De Bodt et al. 2005; Conant and Wolfe 2008; Carretero-Paulet and Fares 2012; Panchy et al. 2016; Soltis and Soltis 2016; Van de Peer et al. 2017). Gene duplicates can be broadly classified into two groups based on the size of the genomic region affected by the duplication. Either they result from whole genome duplications (WGDs), also known as polyploidizations, involving the entire genome and thus affecting all genes in the genome, or they originate form small-scale duplications (SSDs), restricted to small genomic regions and mostly involving one to a few genes. Both WGDs and SSDs are highly prevalent among flowering plants (Van de Peer et al. 2009a, 2017; Vanneste et al. 2014), making them perfect models to study evolution after gene duplication. Although most WGDs are followed by intense fractionation (gene loss) and/or genomic rearrangements, removing much of the duplicated genetic features, successful WGDs can be traced back at the base of main plant lineages (Jiao et al. 2011; Amborella Genome Project 2013), but see also Ruprecht et al. (2017), while more recent WGDs occurred independently in many lineages (Van de Peer et al. 2009a; Vanneste et al. 2014; Soltis and Soltis 2016). For example, in the widely used plant model species *Arabidopsis thaliana*, four WGD events have been detected throughout its evolution (Blanc et al. 2003; Bowers et al. 2003). The most recent ones, namely α and β events, are specific to the Brassicaceae family of rosid eudicots to which *Arabidopsis* belong, whereas the older ones, designated as γ and ɛ WGD events, are specific to the eudicot and angiosperm lineages, respectively (Jaillon et al. 2007; Amborella Genome Project 2013). Likewise, the asterid eudicot *Solanum lycopersicum* (tomato), a model fruit crop, shares the γ and ɛ duplication events with *Arabidopsis* and has undergone a more recent whole genome triplication estimated to have occurred around 64 Ma (Tomato Genome Consortium 2012). Finally, also the monocot *Zea mays* (maize) bears traces of several WGD events, the most recent one dated around 5–12 Ma, after divergence with its close relative *Sorghum bicolor* (Blanc and Wolfe 2004b; Schnable et al. 2009). In turn, SSDs can have different origins, including tandem gene duplication and TE-mediated duplication or retroduplication, the most common one being tandem duplication originating from unequal crossing-over resulting in clusters of linearly arranged genes with no or few intervening gene sequences (Panchy et al. 2016). Together with WGD duplicates, tandem duplicates represent the vast majority of duplicates in plants (Panchy et al. 2016).

Previous studies have reported notable differences in the evolutionary and functional fate of duplicates depending on the mechanism or mode of duplication. For example, genes with certain biological functions (e.g., transcriptional regulation, signal transduction, protein transport, and protein modification) are preferentially retained after WGD, whereas they are rarely retained after SSD, and vice versa (Blanc and Wolfe 2004a; Maere et al. 2005a; Carretero-Paulet and Fares 2012; Rodgers-Melnick et al. 2012; Chen et al. 2013; Jiang et al. 2013; Li et al. 2016; Rody et al. 2017). This patterns seems to be universally true because it has also been observed for fungi and vertebrates (Hakes et al. 2007; Wapinski et al. 2007; Makino and McLysaght 2012). Among the different models proposed to explain such biased pattern of loss and retention of duplicates, only the dosage balance hypothesis is claimed to predict such reciprocity between WGD and SSD duplicates (Freeling and Thomas 2006; Freeling 2009; Birchler and Veitia 2014; Conant et al. 2014). The dosage balance hypothesis states that genomes evolve in such a way that encoded proteins forming part of molecular networks and multiprotein complexes or that involved in multiple steps of biological or regulatory pathways, must remain in optimal balance. It is assumed that WGD duplicates do not upset stoichiometry in the cell because all genes in the genome are duplicated simultaneously. Therefore, WGD duplicates will be preferentially retained, as their loss is expected to lead to a dosage imbalance. Conversely, SSD results in one, or few additional gene copies that are likely to upset dosage balance—at least when part of multiprotein complexes or intricate gene regulatory networks—and result in fitness defects, and thus SSD duplicates are expected to be gradually inactivated and deleted from the genome (Lynch and Conery 2000; Conant and Wolfe 2008; Panchy et al. 2016). However, dosage balance is not indefinitely active, and other forces may be at play to explain longer retention times of duplicates (Conant et al. 2014), including selection on absolute gene dosage if higher expression is selectively beneficial (Hudson et al. 2011; Van de Peer et al. 2017), mutational robustness conferred by genetic redundancy (Gu et al. 2003; Keane et al. 2014), interference in the formation of homomultimeric complexes of paralogs harboring degenerative mutations, that is, dominant negatives (Kaltenegger and Ober 2015), or prolonged opportunity for functional specialization to occur (Lynch and Conery 2000; Conant and Wolfe 2008; Conant et al. 2014; Panchy et al. 2016).

The dosage balance hypothesis predicts that reciprocally retained genes are more constrained to evolve novel or specialized functions in order not to upset the dosage balance. Such a prediction was confirmed among *Arabidopsis* gene families classified as dosage balance sensitive using a modeling approach, which were shown to exhibit stronger sequence divergence (SD) constraints and lower rates of functional and expression divergence (ED) (Tasdighian et al. 2017). In agreement with this, 1) duplicates in *Arabidopsis* and poplar resulting from the relatively recent Brassicaceae- and salicoid-specific WGD events, respectively, display lower divergence in expression than tandem duplicates (Casneuf et al. 2006; Rodgers-Melnick et al. 2012), 2) duplicated genes belonging to functional classes and metabolic pathways that are putatively dosage sensitive based on duplication history exhibited reduced expression variance across species after the shared WGD in the *Glycine* lineage (Coate et al. 2016), and 3) WGD duplicates were found to evolve under stronger purifying selection than contemporary SSD duplicates (Yang and Gaut 2011; Carretero-Paulet and Fares 2012; Rodgers-Melnick et al. 2012). Similar differences between duplicates according to their mechanism of duplication could also be observed in the yeast *Saccharomyces cerevisiae*, with WGD duplicates being functionally less different from one another than SSD duplicates (Hakes et al. 2007; Fares et al. 2013). In contrast, Wang et al. reported that WGD duplicates in *Arabidopsis* and rice show greater divergence in expression than tandem duplicates, although differences in the latter were not found to be significant (Wang et al. 2011).

Some findings referring to the impact of protein–protein interactions (PPIs) on duplicate gene evolution are less readily anticipated by the dosage balance hypothesis. For example, a substantial number of WGD duplicates from *Arabidopsis* have diverged in PPI partners, with conservation declining steadily with the age of the WGD (Guo et al. 2013). Indeed, only a minor fraction of duplicates from the most recent WGD event in *Arabidopsis* involved in PPIs share the same duplication status. The authors claim that the retention of a majority of duplicated gene pairs is no longer explainable by requirements to maintain dosage balance with their interaction partners. Furthermore, although WGD duplicates from *Arabidopsis* and humans display more protein interactions in PPI networks than SSD ones and singletons, differences are only significant for recent duplicates of genes specific to plants or metazoans, respectively (D’Antonio and Ciccarelli 2011; Alvarez-Ponce and Fares 2012). Interestingly, such relationship between centrality in PPI networks and duplicability is inverted in *Escherichia coli*, yeast, worm, and fly (D’Antonio and Ciccarelli 2011). In order to increase our understanding in how PPIs, as well as the mode of duplication, affect gene retention, and the subsequent evolutionary and functional fate of duplicates following WGD and SSD, we here examined a curated data set of WGD and SSD duplicates in *Arabidopsis*, tomato, and maize, a large RNAseq expression compendium with uniquely mapped reads, and an extensive *Arabidopsis* PPI network. Our results point to a key role for PPIs in contributing to dosage balance sensitivity of genes, ultimately helping to explain the biased loss and retention patterns of WGD versus SSD duplicates.

## Materials and Methods

### Delineation of Gene Families and Identification of Gene Duplicates

Gene families and gene duplicates were delineated and identified for *Arabidopsis*, tomato, maize, and 34 additional flowering plant species as previously described (Li et al. 2016), on the basis of a newly PLAZA 3.0 instance (Proost et al. 2015). The workflow ascribes genes to gene families while homologous regions within and between genomes were identified using i-ADHoRe 3.0 (Proost et al. 2012), with 5 as the minimum number of genes required to define a homologous genomic region as collinear (anchor_points 5), 30 as the maximum number of genes between gene pairs to be considered tandem duplicates (tandem_gap 30), and the rest of settings as reported (Van Bel et al. 2012). Duplicates were further classified as block or tandem duplicates depending on whether they were located in collinear regions of the genomes or were found in the same genomic region as clusters of tandemly arranged genes within a maximum of 30 genes apart, respectively.

### Estimates of Synonymous and Nonsynonymous Substitution Rates

For each pair of duplicated genes, codon sequences were aligned with PRANK (version 100701) using the empirical codon model (Kosiol et al. 2007) (setting -codon) to align coding DNA, always skipping insertions (-F). Estimates of synonymous (*K*_s_) and nonsynonymous substitution rates (*K*_n_) were obtained using the CODEML program in the PAML package (v4.8) (Yang 2007) under the GY model with stationary codon frequencies empirically estimated by the F3 × 4 model (Goldman and Yang 1994). To avoid suboptimal estimates because of maximum likelihood entrapment in local maxima, each calculation was repeated five times, and estimates resulting in the better likelihood were used. Also, in order to reduce the influence of genetic redundancy and of synonymous substitutions saturation from old duplicates, duplicates with a *K*_s_ lower than 0.05 and higher than 5, respectively, were discarded from further study (Vanneste et al. 2013).

### RNAseq Compendia and Expression Measures

The *Arabidopsis* RNAseq expression compendium was downloaded from Cornet 3.0 and consists of precompiled expression data sets grouping a total 56 experiments (supplementary table S1, Supplementary Material online) (Van Bel and Coppens 2017). The tomato and the maize RNAseq expression compendia were, in turn, taken from the NCBI’s Sequence read archive and comprise 84 and 77 different experiments, respectively (supplementary tables S2 and S3, Supplementary Material online). Experiments included a mixture of stress conditions, tissue samples, and developmental stages. The three expression data sets were analyzed using the following pipeline: Trimmomatic 0.30 (Bolger et al. 2014) was first used to perform quality filtering and adaptor removal of the sequencing reads. The reads were then mapped using GSNAP 2015-06-23 (Wu et al. 2016), only retaining uniquely mapped reads. Gene counting was subsequently done using Htseq-count 0.6.1 (Anders et al. 2015), and the resulting counts further transformed to counts per million using EdgeR 3.12.1 (Robinson et al. 2010). To ensure data quality, low expression filtering was performed by removing genes with a sum expression count over all conditions lower than two times the number of total conditions. In total, 19,318 *Arabidopsis*, 19,495 tomato, and 23,164 maize genes were uniquely mapped. The ED between duplicated genes was defined as the relative number of conditions in which only one of the duplicates is detected (*C*_1_ and *C*_2_), divided by the total number of conditions in which they are detected (*C*).

$$\mathrm{ED}=\frac{C_{1}+C_{2}}{C}.$$

This measure considers the number of conditions in which the duplicates are expressed and reduces differences due to the combination of different experiments. A measure of 0 means that both duplicates are always expressed in the same conditions. A measure of one means that the duplicates were never detected together.

### PPI Data

A compendium of PPIs in *Arabidopsis* was constructed combining the following sources: BioGRID 3.4 (Chatr-Aryamontri et al. 2013), *Arabidopsis* Interactome (Arabidopsis Interactome Mapping Consortium 2011), MIND (Jones et al. 2014), CORNET 3.0 (only experimentally validated interactions) (De Bodt et al. 2012), STRING v9.1 (only category binding) (Franceschini et al. 2013), EVEX (http://evexdb.org/) (Van Landeghem et al. 2013) (only category binding), and a data set resulting from transporter associated with antigen processing experiments assembled from literature (Takahashi et al. 2008; Pauwels et al. 2010; Van Leene et al. 2010; Bassard et al. 2012; Eloy et al. 2012; Antoni et al. 2013; Cromer et al. 2013; Di Rubbo et al. 2013; Heijde et al. 2013; Spinner et al. 2013; Cuellar Perez et al. 2014; Fonseca et al. 2014; Gadeyne et al. 2014; Vercruyssen et al. 2014). After removing redundant and self-interactions, we obtained a set of 52,613 interactions for 10,266 proteins. The interaction divergence (ID) between two *Arabidopsis* duplicates was calculated as one minus the retention rate, which in turn was defined as two times the number of interaction partners shared between two duplicates ($I_{1,\;2}$) divided by the sum of total interactions in each of the duplicates ($I_{1}$,$\;I_{2}$).

$$\mathrm{ID}=1-\frac{2I_{1,\;2}}{I_{1}+I_{2}}.$$

In order to categorize tomato and maize duplicates as establishing PPIs or not, *Arabidopsis* PPIs were transferred onto the corresponding orthologous genes in tomato and maize according to the genome-wide gene family classification of these three species together with 34 additional flowering plant species (Li et al. 2016). If at least one interaction was present in one of the *Arabidopsis* genes, all tomato and maize co-orthologous genes in the corresponding gene family were assigned to the category with PPI.

### Functional Enrichment Analysis

Enrichment of Gene Ontology (GO) functional terms was calculated using BINGO 2.44 (Maere et al. 2005b), the *Arabidopsis* gene association file from TAIR (GOC Validating Date: March 31, 2017) and the goslim_plant subset version 1.2 (Gene Ontology Consortium 2015). We used hypergeometric and Fisher’s exact tests with a *P* value threshold of 0.05 after Benjamini and Hochberg (BH) correction for multiple testing (Benjamini and Hochberg 1995).

## Results

### Classification of Gene Duplicates, Expression Data Mapping, and PPIs in *Arabidopsis*, Tomato, and Maize

A total of 5,232, 6,645, and 10,654 pairs of duplicated genes were identified in *Arabidopsis*, tomato, and maize, respectively. Duplicates (i.e., ohnologs or homeologs) located in collinear regions of the genomes were further classified as block duplicates putatively arising from WGD events, whereas duplicates found in a singular genomic region were identified as tandem duplicates, conforming the majority of SSD duplicates (table 1). The duplicates that were marked to be both tandem and block duplicates and the ones that could not be unambiguously assigned to any duplication mode were labeled “unclassified” and discarded from further analysis.

**Table 1:** Distribution of Tandem and Block Duplicates with and without PPIs in *Arabidopsis*, Tomato, and Maize

	Tandem			Block			Unclassified			
	Total	With PPI	Without PPI	Total	With PPI	Without PPI	Total	With PPI	Without PPI	Total
*Arabidopsis*	1,130	396	734	1,919	1,308	611	2,183	1,199	984	5,232
Tomato	1,534	350	1,184	1,077	693	384	4,034	1,519	2,515	6,645
Maize	1,692	262	1,430	3,400	1,884	1,516	5,562	1,524	4,038	10,654

We used an expression data set consisting of a compendium of RNAseq experiments for *Arabidopsis*, tomato, and maize (supplementary tables S1–S3, Supplementary Material online). The reads were uniquely mapped and low expression filtering was applied to ensure data quality. Unlike previous studies, where mostly microarray expression data with a low detection rate of duplicates were used (Casneuf et al. 2006; Ganko et al. 2007; Wang et al. 2011; Rodgers-Melnick et al. 2012; Jiang et al. 2013), RNAseq expression data with unique mappings allowed us to individually detect most of the duplicated genes in a pair. In contrast, ATH1 *Arabidopsis* microarrays lacked probes to detect both genes in 38% of duplicate pairs, likely because of cross-hybridization (supplementary table S4, Supplementary Material online). After unique mapping of the reads, expression values were found for both duplicated genes in 63%, 44%, and 48% of *Arabidopsis*, tomato, and maize pairs, respectively. We observed significantly more block duplicates in which both genes in the pair were represented in terms of expression data (79–84%) than tandem duplicates (27–33%) (hypergeometric tests *P* values: *Arabidopsis P *=* *1.16 × 10^−183^, tomato *P *=* *2.20 × 10^−55^, and maize *P *=* *4.72 × 10^−84^) (supplementary fig. S1 and supplementary table S5, Supplementary Material online). Tandem duplication is a continuously on-going process, and very recent duplicates are expected to show little or null SD, likely resulting in the observed higher number of young tandem duplicates without unique expression read mapping.

Finally, we assembled a compendium of *Arabidopsis* PPIs based on small- and large-scale experiments. A total of 2,903 *Arabidopsis* duplicates were found as involved in PPIs. Tomato and maize duplicates were further categorized as involved in PPIs or not by projecting PPI data from *Arabidopsis* duplicates onto their corresponding orthologous genes in these two species, using the genome-wide gene family classification of 37 species of flowering plants (Li et al. 2016). A total of 2,562 and 3,670 pairs of duplicates with PPIs in at least one member of the pair were predicted in tomato and maize, respectively (table 1).

### Block Duplicates Evolve Slower than Tandem Duplicates

Previous studies on *Arabidopsis* and poplar duplicates supported that the mechanism of duplication resulted in differential constraints to evolve, with WGD duplicates generally evolving under stronger purifying selection (Yang and Gaut 2011; Carretero-Paulet and Fares 2012; Rodgers-Melnick et al. 2012) or displaying lower divergence in expression than tandem ones (Casneuf et al. 2006; Rodgers-Melnick et al. 2012). In order to test, and eventually confirm these observations with our three-species data set, we calculated measures of divergence at the level of sequence (SD) and expression (ED) for each of the duplicate pairs in all three species. The rates of nonsynonymous substitutions (*K*_n_), resulting in amino acid changes, were used as estimates of SD between duplicates and also, indirectly, as a proxy for functional divergence (Fares et al. 2013). In turn, ED was calculated as the relative number of conditions in which only one of the duplicates is detected.

**Fig. 1.**
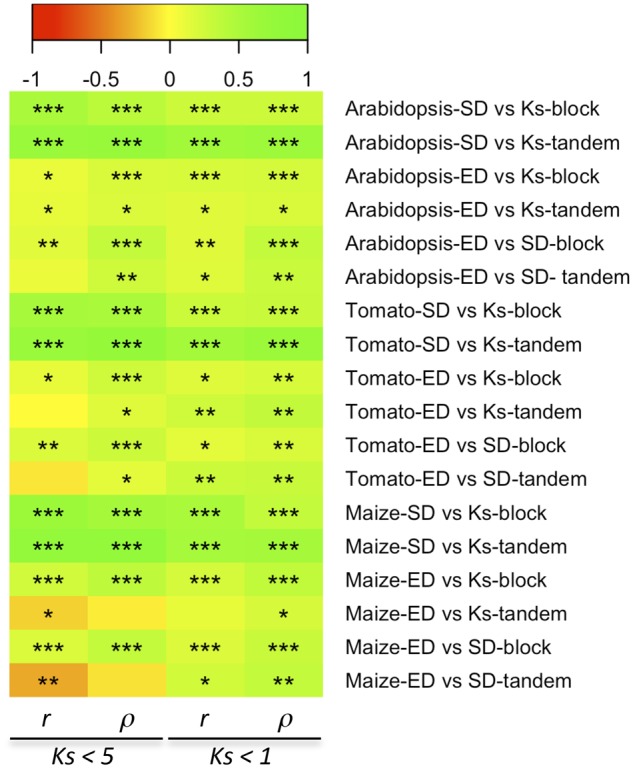
—Heat map of pairwise correlation analysis among *K*_s_, SD (*K*_n_), and ED in *Arabidopsis*, tomato, and maize duplicates partitioned by mechanism of duplication (block vs. tandem). Pearson’s (*r*) and Spearman’s rank (*ρ*) correlation coefficients resulting from comparing subsets of duplicates with *K*_s_ < 5 or *K*_s_ < 1 are colored according to the legend, and the significance level (***, <10*x* − 10; **, <10*x* − 5; *, <0.05) of the associated *P* values are shown.

First, we examined the relationship among *K*_s_, SD, and ED, as well as the putative influence of the mechanism of duplication, by performing pairwise Pearson and Spearman rank correlation tests among these variables for duplicates in all three species partitioned by mechanism of duplication. It had been previously suggested that correlation of ED with *K*_s_ only occurred among younger duplicates (Wang et al. 2011). To account for this, we generated a second subset of younger duplicates restricted to those with estimates of *K*_s_ < 1. In all three species and for both modes of duplication and subsets of duplicates, we found a strongly significant positive correlation between *K*_s_ and SD both through Pearson and Spearman rank tests (fig. 1 and supplementary table S6, Supplementary Material online). With respect to ED, a positive correlation with *K*_s_ was only found among block duplicates, although *r* were generally pretty low (supplementary table S6, Supplementary Material online). In turn, among tandem duplicates, only a marginally significant positive correlation was found between *K*_s_ and ED in *Arabidopsis*, being nonsignificant in tomato, or even marginally negative in the case of maize (fig. 1 and supplementary table S6, Supplementary Material online). Similar results were obtained between SD (*K*_n_) and ED, with only block duplicates displaying a significant positive correlation, whereas tandem ones showed no significant correlation, or a negative one as in the case of maize (fig. 1 and supplementary table S6, Supplementary Material online). Interestingly, although Spearman’s rank tests generally resulted in better correlation coefficients and *P* values, no significant negative correlation was found for any subset of duplicates and comparison performed. Similarly, we found no significant negative correlation in any comparison when we restricted our analysis to duplicates showing *K*_s_ < 1. Taken as a whole, these results seem to indicate that the occurrence of species-specific outlier duplicates with high *K*_s_ values would be altering the linear relationship between SD and ED found for younger duplicates and support previous observations about the heterogeneous relationship between SD and nucleotide substitutions (Wang et al. 2011).

**Fig. 2.**
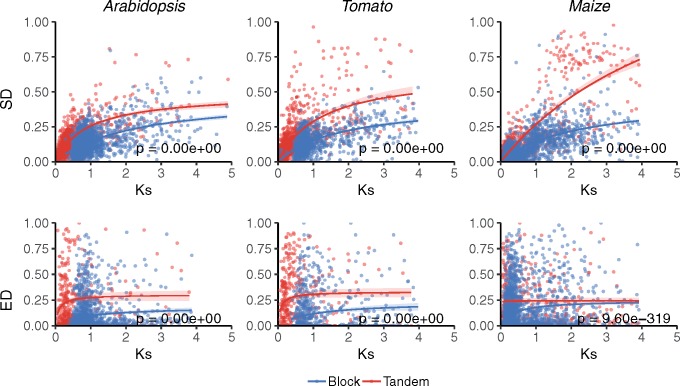
—Evolution of sequence (SD) and expression (ED) divergence of tandem and block duplicates in *Arabidopsis*, tomato, and maize. SD (upper panels) and ED (lower panels) plotted as a function of *K*_s_. For every species, Michaelis–Menten-type saturation curves were fit to SD or ED values of tandem and block duplicates independently. Ninety five percent confidence regions are indicated as colored areas around the corresponding curves. The *P* values on the plots result from *F*-tests for fitting two Michaelis–Menten-type curves independently for tandem and block versus one curve to the combined data set of all duplicates (data not shown).

We further studied the impact of the mechanism of duplication on the evolution of SD and ED over time, using *K*_s_ as a proxy of evolutionary time. As synonymous substitutions do not result in amino acid changes, they are not supposed to impact the function and/or structure of the resulting encoded protein, consequently accumulating throughout evolution in a (nearly) neutral manner. Because of the low coefficients obtained in the correlation analysis, especially between ED and *K*_s_ or *K*_n_, together with the weak, negative, or nonlinear relationship observed in some species and subsets of duplicates, linear regression did not seem the most appropriate function to model the evolution of SD and ED of duplicates. Furthermore, saturation at *K*_s_ values >1 caused by the gradual accumulation of multiple substitutions at the same site over time is not fully corrected for by current evolutionary models and may lead to spurious results (Vanneste et al. 2013). Therefore, we opted for Michaelis–Menten type saturation curves, which had already been proven successful (Tasdighian et al. 2017) in modeling *K*_s_ saturation for old(er) duplicates. Assuming functional redundancy at the time of duplication (i.e., ED and SD should be 0), we model the putative impact of the mechanism of duplication over evolution by plotting our estimates of ED and SD between duplicates as a function of *K*_s_, and fitting independent Michaelis–Menten type saturation curves to tandem and block duplicates. Significance of the differences of the variances between subsets of duplicates was assessed through *F*-tests for testing the hypothesis of fitting two curves independently versus a simpler nested model in which one curve was fitted to the combined data set. As shown in figure 2, ED and SD of *Arabidopsis*, tomato, and maize block duplicates putatively arising from WGD events were consistently found to diverge significantly slower over time than tandem duplicates.

**Fig. 3.**
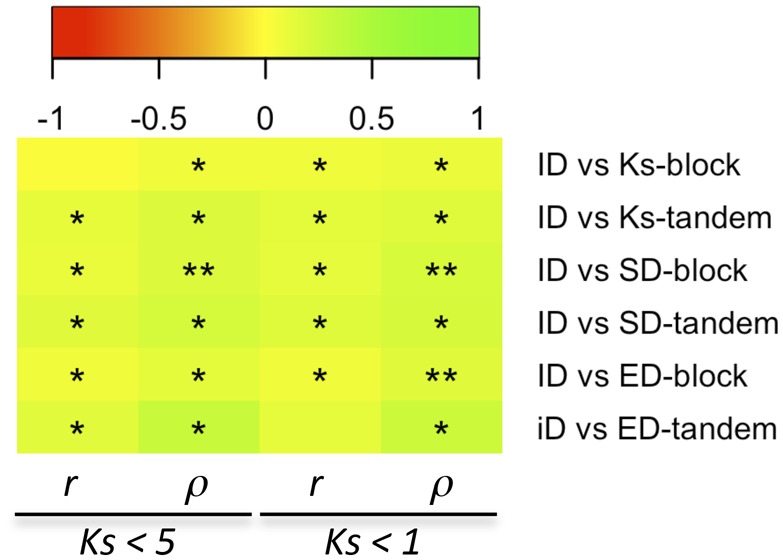
—Heat map of correlation analysis between *K*_s_, SD (*K*_n_), and ED versus ID in *Arabidopsis*, partitioned by mechanism of duplication (block vs. tandem). Pearson’s (*r*) and Spearman’s rank (*ρ*) correlation coefficients resulting from comparing the subsets of duplicates with *K*_s_ < 5 or *K*_s_ < 1 are colored according to the legend, and the significance levels (***, <10*x* − 10; **, <10*x* − 5; *, <0.05) of the associated *P* values are shown.

**Fig. 4.**
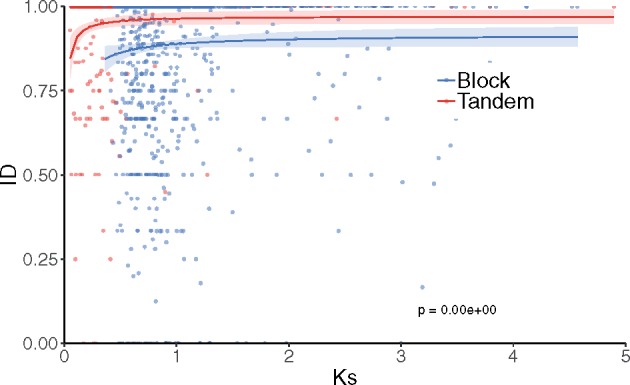
—Evolution of ID of *Arabidopsis* tandem and block duplicates. ID for pairs of *Arabidopsis* duplicates plotted as a function of *K*_s_. Michaelis–Menten-type saturation curves were fit to ID values of tandem and block duplicates independently. Ninety five percent confidence regions are indicated as colored areas around the corresponding curves. The *P* values on the plots result from *F*-tests for fitting two Michaelis–Menten-type curves independently for tandem and block versus one curve to the combined data set of all duplicates (data not shown).

We next explored whether the mechanism of duplication could also be constraining the evolution of divergent PPI partners using measures of ID between *Arabidopsis* duplicated genes. We restricted our analysis to *Arabidopsis*, for which we had assembled a compendium of experimentally determined PPI data. ID was calculated as 1 minus the retention rate, defined as the number of interaction partners shared between two duplicates divided by the sum of unique interaction partners of both duplicates. In order to reduce the noise due to the high rate of false negatives (i.e., not all proteins have experimental PPI data), ID was only calculated for duplicates in which one of the duplicates has at least four PPIs and the other duplicate at least one PPI. Seven hundred and eighty eight pairs were found to be above this cutoff. There are more block duplicates (23%) with more than half of the interaction partners conserved, compared with only 6% for tandem duplicates (Fisher’s exact test: *P *=* *1.2 × 10^−^^8^). We also found more tandem duplicates without any shared interaction partners (48%) than block duplicates (30%) (Fisher’s exact test: *P *=* *2.3 × 10^−^^2^). Correlations between ID and *K*_s_ or ID and *K*_n_ were positive and generally significant, although only marginally, especially in the latter. The linear relationship between ID and *K*_s_ or by *K*_n_ is weak, as reflected by the low coefficients obtained (fig. 3 and supplementary table S7, Supplementary Material online). A marginally significant positive correlation was also found between ID and ED (fig. 3 and supplementary table S7, Supplementary Material online). Finally, we plotted ID as a function of evolutionary time and fitted independent Michaelis–Menten curves to block and tandem duplicates. The former appeared to be significantly more constrained to gain or loss different PPI partners than the later, an effect that persists over time (fig. 4). Our analyses were replicated using different cutoffs to assign a pair to the category with PPI (from at least one up to 14 interaction partners in one of the duplicates), always resulting in significant differences between tandem and block duplicates (supplementary fig. S2, Supplementary Material online).

### Duplicates with PPIs Are More Constrained to Evolve Divergent Functions

**Fig. 5.**
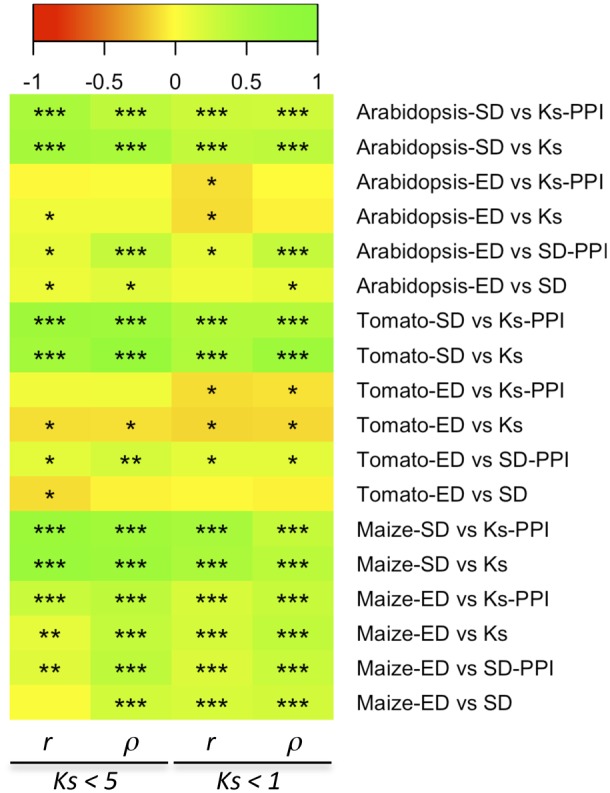
—Heat map of pairwise correlation analysis between *K*_s_, SD (*K*_n_), and ED in *Arabidopsis*, tomato, and maize duplicates partitioned by PPI category (without PPI vs. with PPI). Pearson’s (*r*) and Spearman’s rank (*ρ*) correlation coefficients resulting from comparing the subsets of duplicates with *K*_s_ < 5 or *K*_s_ < 1 are colored according to the legend, and the significance levels (***, <10*x* − 10; **, <10*x* − 5; *, <0.05) of the associated *P* values are shown.

To investigate the putative impact of PPIs on the functional and evolutionary divergence of duplicates, we first examined pairwise correlations among *K*_s_, SD, or ED between duplicates from all three species, partitioned by the PPI category to which the duplicate belongs to (i.e., duplicates without PPI vs. duplicates with PPI), and for two subsets of duplicates (with *K*_s_ < 5 and *K*_s_ < 1). Both Pearson and Spearman rank tests showed a strongly significant positive correlation between *K*_s_ and *K*_n_ in all three species for both PPI categories and subsets of duplicates (fig. 5 and supplementary table S8, Supplementary Material online). In turn, correlation between *K*_s_ and ED was generally low, nonsignificant, or even negative such as in the case of tomato duplicates without PPIs (fig. 5 and supplementary table S8, Supplementary Material online). When we restricted our analysis to the subset of duplicates with *K*_s_ < 1, negative correlations between *K*_s_ and ED could also be detected among tomato duplicates with PPIs, as well as for both *Arabidopsis* duplicates with and without PPIs. In turn, *K*_n_ between duplicates with PPIs showed significant positive correlation with ED in all three species, especially in Spearman rank tests and for duplicates with *K*_s_ < 1. Among duplicates without PPIs, correlation between SD and ED was found to be not significant, or only marginally positive or negative in *Arabidopsis* and tomato, respectively (fig. 5 and supplementary table S8, Supplementary Material online).

**Fig. 6.**
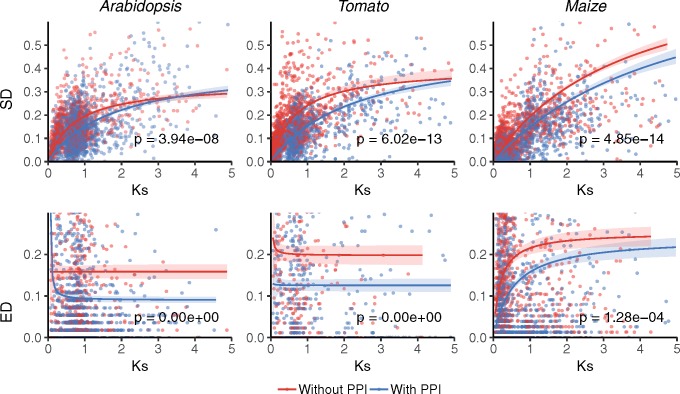
—Evolution of sequence (SD) and expression (ED) divergence of duplicates with and without PPI in *Arabidopsis*, tomato, and maize. SD (upper panels) and ED (lower panels) plotted as a function of *K*_s_. For every species, Michaelis–Menten-type saturation curves were fit to SD or ED of duplicates with and without PPIs independently. Ninety five percent confidence regions are indicated as colored areas around the corresponding curves. The *P* values on the plots result from *F*-tests for fitting two Michaelis–Menten-type curves independently for duplicates with or without PPIs versus one curve to the combined data set of all duplicates (data not shown). In order to improve the interpretability of the results, the *y* axes were truncated at 0.6 and 0.3 for SD and ED, respectively.

Next, we further examined the putative influence of establishing PPIs in the evolution of ED and SD over time, by plotting our estimates of ED and SD between duplicates with and without PPIs as a function of *K*_s_, and fitting independent Michaelis–Menten type saturation curves to each subset of duplicates. As can be observed in figure 6, ED and SD evolve significantly slower in duplicates with PPIs than in duplicates without PPIs in all three species, suggesting the occurrence of PPIs constraints the evolution of duplicates at the expression pattern and sequence level. This constraint generally seems to persist over long evolutionary times, although this may be obscured in the plots due to the low number of duplicates in the upper *K*_s_ region. The constraint on duplicates evolution imposed by PPIs appears to be dependent on the actual number of PPI partners, as reflected their significant negative correlations with SD (Pearson correlation tests: tandem *r* = −0.096, *P *=* *3.4 × 10^−^^3^; block *r* = −0.18, *P *=* *9.7 × 10^−16^) and ED (Pearson correlation tests: tandem *r* = −0.19, *P *=* *3.4 × 10^−^^4^; block *r* = −0.16, *P *=* *3.7 × 10^−10^) of *Arabidopsis* duplicates.

### Block and Tandem Duplicates with PPIs Evolve Slower than Their Counterparts without PPIs

**Fig. 7.**
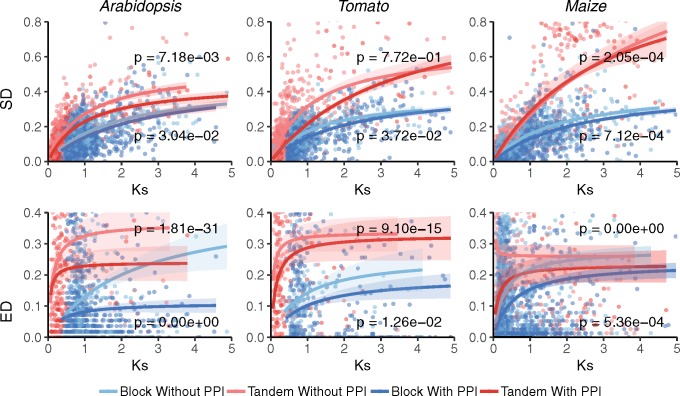
—Evolution of sequence (SD) and expression (ED) divergence of tandem and block duplicates with and without PPIs in *Arabidopsis*, tomato, and maize. SD (upper panels) and ED (lower panels) plotted as a function of *K*_s_. For every species, Michaelis–Menten-type saturation curves were fit to SD and ED values of tandem or block duplicates with and without PPIs independently. Ninety five percent confidence regions are indicated as colored areas around the corresponding curves. The *P* values on the plots result from *F*-tests for fitting two Michaelis–Menten-type curves independently for duplicates with PPIs and without PPIs within each duplication mode versus one curve to the combined data set of all duplicates of each duplication mode (data not shown). In order to improve the interpretability of the results, the *y* axes were truncated at 0.8 and 0.4 for SD and ED, respectively.

With the aim of exploring the interplay between the occurrence of PPIs and the mechanism of duplication in the evolution of ED and SD between duplicates, we plotted estimates of ED and SD for pairs of *Arabidopsis*, tomato, and maize duplicated genes over *K*_s_ by separately partitioning tandem and block duplicates with and without PPIs, and fitted independent Michaelis–Menten type saturation curves to each subset of duplicates. We then performed *F*-tests for fitting two Michaelis–Menten-type curves independently for either tandem or block duplicates with and without PPIs versus one curve to the combined data set of duplicates of each kind (fig. 7). Eleven out of 12 *F*-tests resulted in significant differences in ED or SD between both tandem and block duplicates with and without PPIs (fig. 7). The general picture that emerges is that of duplicates without PPIs displaying faster rates of ED and SD evolution than their counterparts with PPIs.

We next investigated the distribution of PPIs between modes of duplication (table 1). In all three species, PPIs were found to be strongly overrepresented among block duplicate genes (Fisher’s exact tests with BH correction: *Arabidopsis P *=* *3.07 × 10^−37^, tomato *P *=* *2.13 × 10^−49^, and maize *P *=* *5.13 × 10^−116^), whereas underrepresented among tandem ones (Fisher’s exact test with BH correction: *Arabidopsis P *=* *5.25 × 10^−11^, tomato *P *=* *6.68 × 10^−^^8^, and maize *P *=* *2.53 × 10^−48^) (table 1). However, the average number of PPI partners of *Arabidopsis* tandem (6.094) and block duplicates (6.300) did not show significant differences (*t*-test: *P *=* *0.541), which allows to discard the possibility that the differences observed above could be due to differences in the average number of PPI partners between duplication modes.

**Fig. 8.**
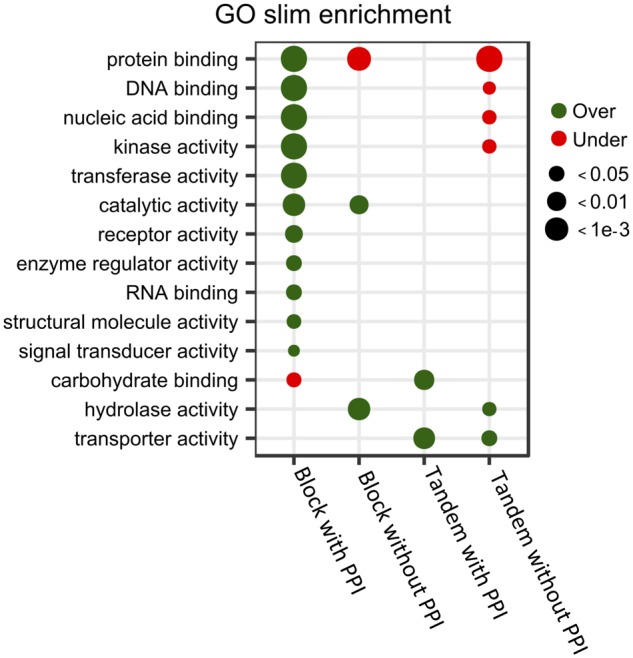
—Functional enrichment analysis of block and tandem duplicates with and without PPI. Enrichment analysis of GO molecular functions belonging to the plant GO slim category for *Arabidopsis* block and tandem duplicates with and without PPI. Only experimentally validated GO annotations were considered. GO terms significantly under- and over-represented (*P* value < 0.05 hypergeometric test with BH correction) are plotted.

Finally, we examined whether PPIs could be also influencing the expected reciprocal pattern of enrichment in GO molecular functions between modes of duplication in *Arabidopsis* (Blanc and Wolfe 2004a; Maere et al. 2005a; Carretero-Paulet and Fares 2012; Rodgers-Melnick et al. 2012; Chen et al. 2013; Jiang et al. 2013; Li et al. 2016; Rody et al. 2017). Block duplicates with PPI were enriched for GO terms associated with binding (protein, nucleic acid, DNA, and RNA), kinase activity (catalytic, transferase), signal transduction/receptor activity, most of which were found as showing no changes or being significantly underrepresented among tandem duplicates with and without PPIs, respectively (fig. 8). This pattern of enrichment contrasted with that of block duplicates without PPIs, where only catalytic activity was similarly overrepresented, together with hydrolase activity, which also popped up as strongly enriched. In turn, tandem duplicates were enriched for transporter activity, with carbohydrate binding and hydrolase activity also found as specifically enriched among those with or without PPIs, respectively.

## Discussion

Here, we have studied the impact of the mechanism of duplication and of PPIs on the evolutionary and functional fate of gene duplicates in three angiosperm plants with different histories of SSD and WGD. By using uniquely mapped RNAseq compendia, we were able to detect the majority of the duplicates in a more robust and reliable way compared with previous studies using microarray data (Casneuf et al. 2006; Ganko et al. 2007; Wang et al. 2011; Rodgers-Melnick et al. 2012; Jiang et al. 2013), although there is still some room for improvement to detect young tandem duplicates in the lower *K*_s_ regions. Furthermore, we assembled a massive compendium of PPI data in *Arabidopsis* and tried to overcome the lack of experimental PPI data in other plant species by projecting our *Arabidopsis* PPI network onto the corresponding orthologs in tomato and maize, with the purpose of categorizing them as establishing PPIs or not. Although orthologous proteins in different species may have evolved divergent functions, including the gain and loss of specific interaction partners, we followed the conservative approach of transferring PPI data between gene families, instead of individual genes. Although this methodology is not perfect and it is likely to result in a high degree of noise, this is not expected to affect SSD or WGD duplicates differently, introducing a bias in our observations.

Our results support contrasting evolutionary dynamics of functional and evolutionary divergence between block and tandem duplicates in all three species, which are likely reflecting their differential contribution to evolutionary innovation and adaptation. Block duplicates consistently diverge slower in terms of SD and ED, indicating stronger purifying selection to evolve novel or divergent protein functions, expression domains or PPI partners, respectively, that may upset dosage balance with other partners of the affected networks. These differences are likely related to the different mutational mechanisms of each mode of duplication; although WGD duplicates entire genes including cis-regulatory regions, SSD often results in incomplete duplication of the gene owing to the random nature of DNA breakage and recombination (Casneuf et al. 2006; Zou et al. 2009). Furthermore, low or null correlations generally observed between ED and nucleotide substitution rates at the level of coding sequences are likely related to the fact that changes in gene expression patterns also rely on changes in promoter or UTR regions (Wang et al. 2011). Similarly, ID showed stronger constraints to evolve among *Arabidopsis* block than tandem duplicates. This pattern did not seem to originate from differences in the average number of PPIs between modes of duplication, as these were not found to be significant as previously noted in *Arabidopsis* (Carretero-Paulet and Fares 2012) and yeast (Hakes et al. 2007).

Although the slower evolution of block duplicates is anticipated by the dosage balance hypothesis, it also raises the question of the biological and evolutionary significance of WGD or polyploidy. The paucity of successful paleopolyploidy events in extant species suggests that polyploidy is usually an evolutionary “dead end” (Van de Peer et al. 2009b; Mayrose et al. 2011; Van de Peer et al. 2017). However, at specific times in evolution, organisms that underwent and survived WGDs might have had some adaptive advantage over their diploid progenitors, eventually contributing to 1) evolutionary diversification and increase in biological complexity (Van de Peer et al. 2009b; Soltis and Soltis 2016, 2009; Van de Peer et al. 2017), as supported by the polyploidy events observed at the base of main plant lineages (Jiao et al. 2011; Amborella Genome Project 2013), but see also Ruprecht et al. (2017) and 2) successful adaptations under periods of extreme environmental stress and/or fluctuations, as suggested by the wave of lineage-specific WGD events observed in angiosperms around the time of the Cretaceous-Paleogene (K-Pg) extinction event (Fawcett et al. 2009; Van de Peer et al. 2009a, 2017; Vanneste et al. 2014). It has been argued that dosage balance selection against functional specialization of block duplicates might be limiting the role of polyploidy on promoting evolutionary change (Tasdighian et al. 2017). However, dosage balance constraints are expected to fade away or change over time (Conant et al. 2014), and thus should be viewed as the primary force driving the retention of duplicates shortly after duplication. Block duplicates retained over longer times may provide with prolonged opportunity for neutral subfunctionalization via the Duplication–Degeneration–Complementation model to occur (Force et al. 1999; Conant and Wolfe 2008; Fares et al. 2013). Subfunctionalization also paves the way for subsequent adaptive evolution under positive selection of novel functions (neofunctionalization) or improvement of ancestral secondary functions (subfunctionalization via the Escape from Adaptive Conflict) (He and Zhang 2005; Conant and Wolfe 2008; Des Marais and Rausher 2008; Panchy et al. 2016). Furthermore, the probabilities of rewiring duplicated networks formed by multiple connected proteins into entire novel complex metabolic, regulatory, or developmental pathways increase if all genes involved duplicate together by means of WGD and evolve synchronously novel or specialized subfunctions, such as interactions partners or expression domains. This way, WGD duplicates originally retained neutrally through requirements to maintain dosage balance, can contribute to the complex adaptive changes at the genomic level and the phenotypic plasticity required in the face of events of evolutionary radiation or ecological challenge.

Tandem duplicates are more likely to upset dosage balance, in special when connected with other proteins. Their retention in the short term will depend on the cost associated with the maintenance of additional gene copies. The faster divergence rates observed for tandem duplicates in all three species may thus reflect the rapid acquisition of novel or specialized functions in order to compensate this cost; otherwise, they are expected to be lost by means of nonfunctionalization or pseudogenization (Lynch and Conery 2000). This, together with across-species differences observed in correlation patterns between ED and *K*_s_ or *K*_n_ for young and old tandem duplicates might suggest their involvement in rapid adaptations to local environmental stimuli, which is in turn supported by species-specific enrichments commonly observed for tandem duplicates in functional categories related to response to stress or secondary metabolism (Hanada et al. 2008; Denoeud et al. 2014; Panchy et al. 2016). Long-term retention of specific duplicates may also result from selection on the absolute dosage of certain gene products, that is, the higher concentration of an enzyme may result in the higher metabolic flux in the cell of the corresponding biochemical pathway (Bekaert et al. 2011; Hudson et al. 2011). This selection is also expected to operate differently on block and tandem duplicates. In pathways where increases in the absolute dosage of a single enzyme have no effect on the resulting metabolic flux, WGDs can provide such a flux increase by duplicating all its components at once (Bekaert et al. 2011). In contrast, enzymes that are working independently or that provide a bottleneck in the pathway could take advantage of a SSD (e.g., hexose transport in yeast) (Sugino and Innan 2006; Arakaki et al. 2011).

Functional and evolutionary divergence of *Arabidopsis*, tomato, and maize duplicates also appeared to be constrained by the involvement of the encoded protein in PPIs, as revealed by the significant slower rates of evolutionary change in terms of SD and ED of duplicates with PPIs. These constraints are dependent on the actual number of PPI partners, as reflected by the low, although significant, negative correlations with SD and ED in both *Arabidopsis* block and tandem duplicates, that is, the higher the number of PPI partners, the higher the constraint for duplicates to diverge. Regions of the protein involved in PPI interactions, that is, PPI interfaces, are conserved through negative purifying selection, which is expected to limit amino acid changes (Lovell and Robertson 2010). Therefore, a given protein involved in multiple PPI interactions is expected to show a reduced number of sequence regions available for evolutionary change to occur without disrupting PPI interfaces, thus resulting in the observed increased selective constraint to diverge. These observations are in agreement with duplicates involved in physical protein–protein, or other molecular or genetic, interactions evolving under stronger purifying selection, because functional divergence of a connected protein is more likely to disrupt the stoichiometry of the affected biological network (Freeling and Thomas 2006; Freeling 2009; Birchler and Veitia 2014; Conant et al. 2014). Furthermore, the fraction of block duplicates with PPIs is significantly larger than that of tandem duplicates, which may be reflecting the fact that the chance of upsetting dosage balance if lost increases for connected WGD duplicates.

Our results also supported PPIs as imposing stronger selective constraints independently of the duplication mode, that is, both block and tandem duplicates with PPIs show slower rates of ED and SD evolution than their counterparts without PPIs. Our functional enrichment analysis further revealed GO molecular functions commonly reported in the literature as associated with dosage sensitive functional classes, that is, transcriptional regulation, development, and signaling (Blanc and Wolfe 2004a; Maere et al. 2005a; Carretero-Paulet and Fares 2012; Rodgers-Melnick et al. 2012; Chen et al. 2013; Jiang et al. 2013; Li et al. 2016; Rody et al. 2017), are specifically enriched among *Arabidopsis* block duplicates with PPIs, with the reciprocal pattern being true for tandem duplicates without PPIs. Interestingly, hydrolase enzymatic activity appeared as enriched in both groups of duplicates without PPIs. Therefore, the reciprocal retention pattern predicted by the dosage balance hypothesis (Freeling and Thomas 2006; Freeling 2009; Birchler and Veitia 2014; Conant et al. 2014) can be, at least partially, explained by the enrichment in PPIs of genes involved in biological functions commonly classified as dosage balance sensitive, rather than by the mechanism of duplication itself. However, it must be noted that the generally low correlation coefficients obtained in our analysis, particularly for ED or ID versus *K*_s_ or *K*_n_, are suggesting that other factors, apart from the mechanism of duplication and PPIs, are affecting the functional divergence of duplicates. These additional factors likely include other biological interactions, apart from physical PPIs, in which the gene, or its product, is involved. Assuming constraints of duplicates to functionally diverge throughout evolution are solely based on dosage balance sensitivity, it is tempting to speculate that subsets of duplicates not involved in any interaction or network, that is, functioning in solitary, if any, will evolve under similar selection regimes. However, the current analysis suggests additional species-specific mechanisms not necessarily influencing dosage balance sensitivity may be at play and highlights the complexity of the mechanisms underlying functional divergence of duplicates throughout evolution (Carretero-Paulet and Fares 2012).

In summary, our results support dosage balance constraints of duplicates to functionally diverge as specific properties of genes, rather than associated with specific biological functions, and resulting from their overall involvement in different kinds of biological interactions and networks. Of these, we have shown the prominent role played by PPIs in explaining differential dosage balance sensitivity and subsequent duplicate retention and contribution to evolutionary innovation and adaptation between modes of duplication. Current progresses on systems biology approaches integrating high-throughput-omics data, together with the development of evolutionary simulation computational frameworks, will help to unravel the contribution of relative dosage balance sensitivity to explain gene evolution after duplication with respect to other models proposed, including absolute dosage balance, functional specialization through neo- or sub-functionalization, mutation robustness, or paralog interference.

## Supplementary Material

Supplementary data are available at *Genome Biology and Evolution* online.

## Supplementary Material

evz156_Supplementary_Data
