# Supplementary material for: The Evolution of Gene Duplicates in Angiosperms and the Impact of Protein–Protein Interactions and the Mechanism of Duplication
Source: Genome Biol Evol. 2019 Jul 31;11(8):2292–305. doi: 10.1093/gbe/evz156 (PMC6735927; doi:10.1093/gbe/evz156)
Supplement: evz156_Supplementary_Data [file evz156_supplementary_data.zip › Suppl legends.docx]

**Legends to Supplementary material**

**Figure S1: Detection of expression for pairs duplicates in Arabidopsis, tomato and maize per duplication modes as a function of Ks.** Please, see also table S5.

**Figure S2: Selection of a minimum number of PPIs to estimate Interaction Divergence (ID).** Significance of differences in ID between tandem and block duplicates (y-axis) plotted as a function of the minimum number of PPIs (x-axis). The table shows the number of block and tandem duplicates that meets the cut-off and the significance of the comparison between block and tandem duplicates. The comparison is always significant (P>0.05) independently of the chosen cut-off value. Based on the first valley, we selected a minimum threshold of four PPIs as cut-off to assign a pair to the category with PPI.

**Table S1: List of RNAseq experiments in the expression compendia for Arabidopsis.**

**Table S2: List of RNAseq experiments in the expression compendia for tomato.**

**Table S3: List of RNAseq experiments in the expression compendia for maize.**

**Table S4: Comparison of detection of expression in Arabidopsis duplicates between RNAseq compendium and ATH1 microarray.** The first table represents the number and percentage of duplicates detected in the RNAseq compendium after filtering. Second table represent the duplicate genes covered by the probes on the ATH1 microarray. The third table shows the comparison between the duplicates for which expression was detected in the RNAseq and the ATH1 microarray.

**Table S5: Summary of pairs of duplicates detected in our compendia of RNAseq data for Arabidopsis, tomato and maize per mode of duplication.**

**Table S6. Pairwise correlation analysis between Ks, SD (Kn) and ED in Arabidopsis, tomato and maize duplicates partitioned by mechanism of duplication (block *vs* tandem).** Pearson’s correlation coefficients (*r*), Spearman’s rank (*ρ*) correlation coefficients, their associated *P* values, and the significance levels (***, < 10x^-10^; **, < 10x^-5^, *, < 0.05) resulting from comparing subsets of duplicates with Ks < 5 or Ks < 1 are shown.

**Table S7. Correlation analysis between Ks, SD (Kn) and ED vs ID in Arabidopsis, partitioned by mechanism of duplication (block *vs* tandem).** Pearson’s correlation coefficients (*r*), Spearman’s rank (*ρ*) correlation coefficients, their associated *P* values, and the significance levels (***, < 10x^-10^; **, < 10x^-5^, *, < 0.05) resulting from comparing subsets of duplicates with Ks < 5 or Ks < 1 are shown.

**Table S8. Pairwise correlation analysis between Ks, SD (Kn) and ED in Arabidopsis, tomato and maize duplicates partitioned by PPI category (without PPI *vs* with PPI).** Pearson’s correlation coefficients (*r*), Spearman’s rank (*ρ*) correlation coefficients, their associated *P* values, and the significance levels (***, < 10x^-10^; **, < 10x^-5^, *, < 0.05) resulting from comparing subsets of duplicates with Ks < 5 or Ks < 1 are shown.
